# Teleorthodontics: Where Are We Going? From Skepticism to the Clinical Applications of a New Medical Communication and Management System

**DOI:** 10.1155/2022/7301576

**Published:** 2022-02-01

**Authors:** Antonino Lo Giudice, Vincenzo Ronsivalle, Pietro Venezia, Rosalia Ragusa, Giuseppe Palazzo, Rosalia Leonardi, Antonio Lazzara

**Affiliations:** ^1^Department of General Surgery and Surgical-Medical Specialties, School of Dentistry, University of Catania, Via S. Sofia 78, 95124 Catania, Italy; ^2^Health Direction of Policlinic Hospital, 95100 Catania, Italy; ^3^Chief Medical Officer of the University Hospital “G. Rodolico-San Marco”, University of Catania, Via S. Sofia 78, 95124 Catania, Italy

## Abstract

Teleorthodontics represents the orthodontic care system involving remote management of orthodontic treatment. Despite skepticism, there are several advantages of including teleorthodontics in the clinical orthodontic practice. In the present review, we discuss the lights and shadows of this new communication healthcare system and its applications in the field of orthodontics that is destined to change the future of our clinical practice. For this purpose, we have provided a point-to-point analysis based on data from the most valuable scientific evidence on this topic. The information and data discussed in the present paper were obtained from the most relevant studies evaluating the performance of teleorthodontics and remote monitoring systems in clinical practice.

## 1. Introduction

Digital technology has revolutionized the conventional communication paradigm and information flow supporting clinicians in the diagnosis, treatment plan strategies, and healthcare management [1]. Concerning patient communication, telemedicine provides the exchange of patient-related information with healthcare providers and/or related organizations using digital technology such as radiographic and photographic records and healthcare-related consultations [1, 2]. Telemedicine boosts communication between specialists, for example, in those cases requiring a multidisciplinary approach, as well as allows patients to easily refer to a specialist for preliminary consultation, clinical follow-up, or treatment monitoring [3]. One of these novel communication paths expands through medical apps for digital tablets or smartphones that showed positive feed-back to enhance the educational system and healthcare services [4]. In this regard, the exchange of healthcare-related information using digital technology has already grown in the last decades; however, the COVID-19 pandemic has boosted telemedicine due to the possibility to provide healthcare consultation in emergency conditions requiring social distance [5].

Orthodontics is not exempt from this scenario, and the term teleorthodontics has been coined to define the orthodontic care system involving remote management of orthodontic treatment [6]. There are several advantages of including teleorthodontics in the clinical orthodontic practice [7], from preliminary patient screening to the reduction of in-office appointments, the latter being beneficial both for clinicians and patients for optimizing daily-time schedule [8]. Also, reducing routine orthodontic appointments is essential during the COVID-19 pandemic, since it avoids the overcrowding of both public and private orthodontic centers. However, skepticism is still prevalent among the orthodontic community with frequent questions such as “How can I treat patients without seeing them?” or “Will remoting systems jeopardize the communication between patient and orthodontists?” and in this case, “If Tele-orthodontics becomes popular, could patients and lay-persons underestimate the role of the clinicians and become confident to do-it-yourself (DIY) or delivered to customer (DTC) orthodontics?”

In this respect, in the present review we aimed to discuss the lights and shadows of this new communication healthcare system and its applications in the field of orthodontics, which is destined to change the future of our clinical practice. For this purpose, we have provided a point-to-point analysis based on data from the most valuable scientific evidence on this topic.

## 2. Literature Review

The information and data discussed in the present paper were obtained from the most relevant studies evaluating the performance of teleorthodontics and remote monitoring systems in clinical practice. Considering the type and novelty of the topic investigated, we proposed a narrative description of the data available from the most relevant studies. Based upon the limited available evidence, we tailored the discussion to provide an overview of the potential benefits, disadvantages, and ethical concerns related to the teleorthodontics and telecommunication systems in this medical field.

## 3. Diagnosis, Treatment Planning, and Patient Screening

**Figure 1:**
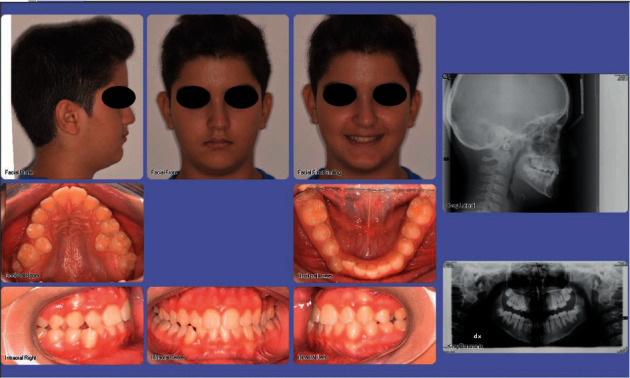
Example of conventional digital diagnostic records formatted in orthodontic software. Clinicians usually make decisions and plan treatment strategies in front of a monitor, taking the time needed to evaluate the patient's characteristics (skeletal, soft-tissue, and dento-alveolar components). These files can also be shared for a multidisciplinary approach.

Conventionally, the diagnostic process in orthodontics is based on the analysis of specific records, such as intraoral and extraoral images, radiographic examinations, and cast stone/digital models [2]. Clinical evaluation, although essential, is not often sufficient to get a comprehensive diagnosis and to generate a detailed problem list, and the treatment planning is generally established after a deep analysis of the diagnostic records acquired [9], including, for example, hard-tissue and soft-tissue cephalometric and profile analysis [10, 11]. Seated at the desk in front of a high-resolution (HD) monitor, with the patient being far away from the dental office, the orthodontist analyzes the diagnostic records and “take its time” to formulate a definitive diagnosis and to develop an adequate treatment plan to correct malocclusion and to satisfy relative patients' concerns (Figure 1) [12]. This process has always been part of the diagnostic workflow, even in the past analogical era; however, it is now emphasized as one of the pillars of teleorthodontics due to the increased resonance of online medical consultations over the last years [13]. Nevertheless, clinical inspection remains an irreplaceable brick of the diagnostic stage since it allows the assessment of specific characteristics that cannot be assessable in front of a liquid crystal display (LCD) screen, and caution should be taken in providing definitive consultation or planning orthodontic treatment without seeing the patient at the chairside [6]. In this regard, clinical inspection and remote patient evaluation represent two different moments of the same diagnostic workflow that take to an accurate treatment plan strategy and successful orthodontic treatment.

Instead, the information retrieved from digital communication systems can be sufficient to satisfy the necessity for a preliminary screening of patients or to identify potential candidates for future orthodontic therapy [14]. For example, through teleorthodontics, clinicians can define a treatment priority list, reducing chairside visits which is cost-effective for patients, especially those living in remote/rural geographic locations, and for orthodontic care providers since it streamlines access to the orthodontic office [14, 15]. In this regard, we suggest clinicians or healthcare manager to enhance Internet communication of their orthodontic brand, for example, by introducing a screening panel to enhance patients' care service.

The recent advances in three-dimensional (3D) imaging have also simplified communication within the orthodontic staff and doctors, since clinicians can use existing patients' digital diagnostic package/records, including digital models, digital scans, photographs, and radiographs, to better communicate and analyze data with colleagues and lab technicians, which is extremely useful when a multidisciplinary approach is required [16].

## 4. Orthodontic Treatment Monitoring

Thanks to the advances in telecommunication and digital technologies, orthodontics is intended to change and optimize the clinical management of treatment time by setting remote patient examinations and shrinking chairside appointments without affecting the effectiveness of the therapy and increasing the benefits-to-cost ratio of the treatment [8, 17]. In this regard, less frequent in-office visits are beneficial for both patients, especially adults, and clinicians [18]. To understand the relevance of this approach, just say that about 15% of scheduled appointments during treatment with fixed appliances are represented by emergency visits, while early interception of developing problems using teleorthodontics systems such as poor oral hygiene, nontracking aligners, broken appliances, or poor compliance may help to reduce treatment times [19, 20]. Reducing the number of face-to-face appointments is also consistent with government restrictions enforced in the COVID-19 pandemic era where both clinicians and healthcare managers are called to design a new ergonomic approach to contribute to the reduction of virus transmission [21].

However, remote patient monitoring has limited applications with fixed appliance treatment since recurrent visits are required for appliance activation [8]. On the contrary, the clinical management of preprogrammed orthodontics with customized appliances usually includes simple visits where the orthodontist “limits to observe” the progress of the planned stage. In this regard, orthodontic treatment based on virtual setup would notably benefit from teleorthodontics since it is possible to eliminate simple visits while maintaining those relevant for the treatment strategies such as stripping, brackets/attachments or auxiliaries placement, and extraction [8, 15, 17]. This would enhance the treatment experience, making orthodontic treatment even more appealing for adults who are seeking invisible and efficient orthodontics [22, 23], but also for clinicians since this would streamline the orthodontic workflow in the office [24].

**Figure 2:**
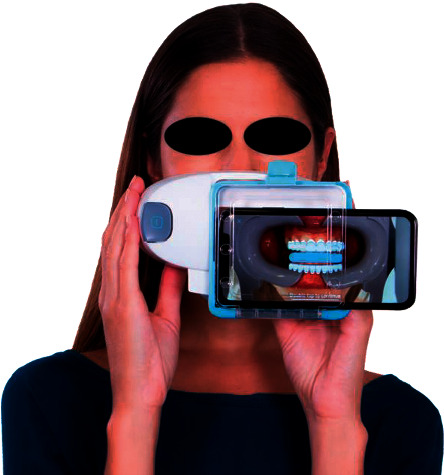
Dental Monitoring system. This smartphone-based app allow patients to scan directly their teeth and provide instantaneous feedback of treatment progress to the clinicians and orthodontic office. The patient insert the smartphone into the ScanBox that allows a more predictable scan procedure.

**Figure 3:**
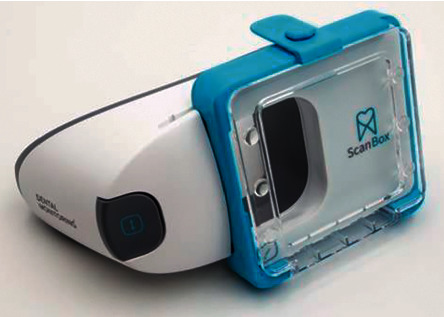
Closed view of the ScanBox shell.

Recently, a smartphone application software for remote monitoring, Dental Monitoring (Dental Mind, Paris, France), has been launched on the market [25]. This is the first SaaS (software as a service) application designed for remote monitoring of dental treatment. The digital technology involved allows the software to track tooth movement by using the images of scanned videos taken by the patients with smartphone cameras (iOS or Android). Briefly, each video scan consisted of three sets of images taken by the patients with the cheek retractors in place. In the first two scans, the patient is asked to turn the head side to side to capture the anterior and lateral segments of dentition, first with the teeth in occlusion and then with both arches slightly apart. In the third set of images, the patient registers the occlusal views of the arches by changing the angle of the smartphone camera while moving the head up and down with the jaws wide open. The package included specific cheek retractors used by patients to facilitate scan acquisition (Figures 2 and 3). The monitoring software makes it possible to track treatment progress between office appointments, prompting clinicians when specific outcomes have been reached such as space closure, space opening, or dental expansion [17]. Thus, this promising software represents a step further in the simple management of clinical emergencies, as it allows users to receive alerts when specific issues are detected, such as a broken appliance, poor hygiene, or gingival recession [17, 25].

The scientific evidence supporting the clinical usage of DM is scarce. However, interesting studies have been recently carried out to address this topic. Hansa et al. [8] found a significant reduction of about 23% of the number of appointments over the treatment duration carried out with clear aligners in the DM group compared with the unmonitored group. These findings are of great clinical relevance since they support the assumption that remote monitoring could decrease chairside time and relative costs to both orthodontic care providers and patients [26, 27]. These data were also confirmed by previous preliminary evidence of the same study group [28]. At the same time, there would be an increase in the frequency of patient monitoring, resulting in a more precise evaluation of treatment by the orthodontist. Instead, no differences were found between the two groups for treatment duration or refinement stages. From a clinical perspective, this means that DM improves the clinical management of the therapy, but, to date, it should not be used with the assumption of reducing the overall treatment time and should be clearly stated to patients.

Since the remote tracking of orthodontic treatment also entails a quantitative assessment of the movement obtained at a specific stage, it is extremely important to validate the DM software by assessing its accuracy in registering and transmitting quantitative data of orthodontic tooth movement. In this regard, a recent well-conducted study [17] compared the software measurements of the intercanine and intermolar widths with those obtained from plaster models (gold standard) and found that they were almost equivalent and within 0.5 mm of tolerance error. Despite the range of error was higher than that reported by the manufacturer, the authors underlined that the quality of the video scans was acceptable for making clinical decisions, considering that in a clinical scenario, clinicians usually rely on visual inspection when evaluating the amount of tooth movement required and achieved (arch expansion in the mentioned study). Accordingly, Kuriakose et al. [29] found that DM accurately assessed the correction of posterior crossbite and that there was no significant difference in intermolar width measurements obtained with DM, digital model, or intraoral examination. Despite the promising findings, the literature is extremely deficient further to this point and is limited to the evaluation of transverse orthodontic movement. Further studies are warmly encouraged to assess the accuracy in registering different types of movement, especially in the posterior region, where patients experience some difficulties in the video scanning procedure [17]. Furthermore, future studies comparing the clinical outcomes (occlusal, esthetic, and functional factors) obtained in different consultation settings (remote versus in-office) are needed to support the effectiveness of teleorthodontics.

The Grin Remote Monitoring Platform (https://get-grin.com/) is another system that offers telemedicine capabilities to orthodontic specialists. In particular, Grin is a comprehensive digital orthodontic platform that enables orthodontists to monitor patients' oral health remotely. It consists of a smartphone app (Grin App) and an intraoral adapter designed to retract the cheeks for a full view of a patient's mouth (Grin Scope). After fixing the Grin Scope to the smartphone, patients can take a self-scan of their teeth while the system registers data and generates an intraoral video. With appropriate instructions, patients can record functional status during video acquisition such as mouth opening, protrusive, and lateral mandibular movements. Afterwards, orthodontists can assess intraoral video via the online doctor portal. This virtual access would allow clinicians to monitor orthodontic treatment at a distance, reducing in-office routine appointments. However, there is no scientific evidence in the literature concerning the usage of this system, and studies conducted in clinical settings are warmly encouraged.

## 5. New Ergonomic Concept: Teleorthodontics and Removable Appliances

In its evolutionary process and young history, orthodontics has gone through several innovations that have significantly contributed to the development and standardization of the treatments. Most of these changes represented the overcoming of previous diagnostic and therapeutic limitations faced by the clinical community and thus derived from an internal demand, among them 3D imaging systems, 3-dimensional radiology, skeletal anchorage, brackets, etc. The advent of clear aligners has been another evolutionary milestone since it has represented the first noteworthy response to an “external” demand for esthetic treatments from the social community. Despite such demand having been influenced by aggressive manufacturers' marketing strategies, treatment with clear aligners should be considered a new patient-centered approach that must take into account not only the final clinical outcome but also the patients' treatment experience [30, 31]. Another actual patient request, especially among adult subjects, is to undergo orthodontic treatment with less frequent in-office visits, meanwhile allowing the specialist to maintain control over their treatment progress. In an increasingly time-conscious world, simple visits for routine checks of the appliance are considered a “waste of time” by patients, and they would prefer to come to the office only for landmark appointments. Moreover, in this scenario, the COVID-19 pandemic has imposed a reduction of the number of face-to-face appointments and meetings in order to contain virus infection [5, 32].

With these notions in mind, a new ergonomic system is necessary to streamline the clinical workflow. In this system, teleorthodontics, remote communication, and remote monitoring technologies are useful tools to keep evaluating clinical progress and manage emergencies while reducing the number of clinical appointments. Along with telecommunication, encouraging the usage of clear aligners over fixed appliances (when supported by appropriate diagnosis and treatment plan strategy) would be cardinal in this process since aligners require less modifications and activations, which reduce the number of visits for debonding, injuries to the oral mucosa, etc. [33–36]. Moreover, an early clinical approach involving (1) adequate screening of patients during growing age and (2) interceptive treatment or orthopaedic/functional treatment of skeletal disharmonies/malocclusion [37] would facilitate orthodontic treatment at the permanent dentition stage, which in turn would simplify the clinical management of the therapy and the number of appointments in the long term [38].

In general, this new ergonomic strategy involves a new mindset, and clinicians are called to leave their comfort zones and change their habits in terms of digitalization of the workflow and usage of removable appliances [21, 32, 39, 40].

## 6. Patient-Orthodontist Relationship: From Psychological Perspective to Ethical Concerns

How do teleorthodontics and remote monitoring affect patient-orthodontist relationships? Can telecommunication decrease patients' perception of orthodontics as a medical treatment? Is teleorthodontics the final incentive, driven by the market, toward “do it yourself” (DIY) or “direct to consumer” (DTC) orthodontics? These are some of the main questions concerning teleorthodontics, in particular, addressed by wise and experienced orthodontists who are conscious that a solid patient-doctor relationship represents the foundation of successful orthodontic treatment and that most of the controversies generally arise from misunderstandings and from a lack of awareness of clear limits between them. In other words, there are two hypothetical risks that feed skepticism among clinicians. Firstly, since teleorthodontics reduces face-to-face visits, it may instill in laypersons the idea that the role of the doctor (orthodontist) is less relevant than previously thought. Such concern is reasonable considering that the orthodontic market is being assaulted by companies that offer at-home impression kits, which allow clients to take their own impressions and receive clear aligners at home at low price, replacing the orthodontists with a “do it yourself” (DIY) concept [40]. In this regard, confusing individuals, seeking less expensive orthodontic treatment could wonder, “Why should I go to orthodontic office if orthodontists already monitor their patients by remote systems, without seeing them face-to-face?” The second risk regards the greater difficulties in establishing a solid and respectful relationship with the orthodontist and telemonitored patients. In this regard, the patient-doctor relationship is the pillar of successful treatment, especially in the management of complex cases or when cooperation is required [41, 42].

To try to answer all these concerns, let us take a step backward to evaluate patients' experiences of teleassisted orthodontics according to the most recent scientific evidence. As a matter of fact, there are only a few studies addressing this topic and investigating dental monitoring software. Hansa et al. [6, 28] found a general positive patient perception and experience using DM, with common benefits mentioned being better communication with the orthodontist, the possibility to provide feedback and to be more conscious of the progress of the treatment, a reduced number of in-office visits, and increased convenience. In this regard, the DM app allows for direct message communication with the orthodontic office, which mitigates the communication problems that may occur with less frequent office visits [25]. Thus, dental monitoring can represent a strength of the orthodontic care service; in fact, from the clinical perspective, it can constitute an improvement in terms of compliance and treatment success, and from the business perspective, it allows to hindering the DTC phenomenon by highlighting the importance of constant medical monitoring during the orthodontic treatment [18, 25].

Nevertheless, the same authors [6, 28] found that 12% of their sample would prefer to have more in-office visits, supporting the assumption that reducing the number of face-to-face appointments may diminish the rapport between doctor and patient [43]. In this regard, malpractice lawsuits may increase if patients feel they are not receiving treatment of a satisfying quality [32]. Some of these patients also experienced difficulties with the management of DM and scan acquisitions. These findings enlarge the discussion on the necessity of targeting the usage of teleassistance according to patients' needs, psychological characteristics, and digital backgrounds. For example, teleorthodontics can be a trump card within a hectic or busy lifestyle environment since patients can feel monitored by the specialist while reducing the time “wasted” at the dental office; instead, teleassistance could have some difficulties in taking root in other areas, often small cities of southern countries, where human contact is still at the basis of relationships, including that between patient and doctor. Instead, concerning digital skills, the usage of dental monitoring should not be a major concern since a large part of the subjects seeking orthodontic treatment are digital natives or fair digital immigrants. However, we warmly encourage further studies to better elucidate how teleorthodontics can fit different types of populations.

## 7. Privacy Issues and Informed Consent Related to Teleorthodontics

Teleorthodontics, form general teleassistance to remote treatment monitoring, involves the transmission of sensitive data such as photographic/radiographic records and anatomical digital files throughout internet-based telecommunication systems. The delivery of healthcare and the storage of medical information on devices such as tablets, mobile phones, and laptop computers raises concerns about the protection of a patient's privacy and the violability of healthcare information. In this regard, orthodontic care providers must obtain signed consent that must be freely given, specific, informed, and unambiguous, following the guidelines of the General Data Protection Regulation European law (EU GDPR) [44, 45]. It should be mentioned that there is a risk of privacy violation when patients' photos, radiographs, and other healthcare-related information are shared using teleorthodontics. Moreover, the consent should also clearly state that a breach and/or loss of electronic communication may also indicate a loss of services offered via teleorthodontics. Another important issue is to inform patients about who will analyze sensitive data and how they will be diffused [46]. One possibility would be to secure images with watermarking and transmit them with encryption or through private networks, based on the guidelines of the American Telemedicine Association [47, 48].

The step further privacy concerns is the patients' consensus to the therapeutic limitations of teleorthodontics or remote monitoring systems. For example, in the case of virtual consultations, patients must be aware that a comprehensive in-office orodental and craniofacial evaluation is essential for orthodontic diagnosis and treatment planning and that the clinical information provided cannot be considered as based on a definitive diagnosis [2]. In the case of usage of dental monitoring system, due to the absence of specific guidelines, the authors of the present paper warmly suggest clinicians to generate an appropriate informed consent form attesting to the willingness of the patient to co-operate and being compliant with the monitoring protocol defined by the clinician or the medical staff.

## 8. Discussion and Conclusion

The clinical perspective of teleorthodontics is virtually endless. Remote consultations and treatment monitoring will enhance the treatment experience as well as treatment efficiency if we take chairside time as reference. Considering the general characteristics of the orthodontic treatment, remote monitoring represents the strong point of clinical application of teleorthodontics. Despite skepticism, patients' feedback seems to be positive with a high percentage of subjects that do not lose the perception of being monitored as a consequence of the less number of in-office visits. However, a significant percentage of subjects still prefer in-office appointments, suggesting that it would be beneficial to introduce this technology gradually into clinical practice according to the economic, social, and psychological characteristics of the patients. In this regard, the key to remote monitoring seems to be the balance between the benefits of in-office visits and direct patient-orthodontist relationships and the convenience (including costs) of remote consultation, based on each individual and each orthodontic office. Further research should be considered using digital diagnostic tools and software in orthodontics.

## Data Availability

Data are available upon reasonable request to the corresponding author.
